# Problems and possibilities of chemistry on dry reagent carriers

**DOI:** 10.1155/S1463924681000199

**Published:** 1981

**Authors:** Jerome Greyson

**Affiliations:** Blood Chemistry Laboratory Ames Division Miles Laboratories Inc. Elkhart Indiana USA


### Problems and possibilities of chemistry on dry reagent carriers*

**Jerome Greyson**
*Blood Chemistry Laboratory, Ames Division, Miles Laboratories Inc., Elkhart, Indiana, USA.*

### Introduction

It is apparent to all working in the field of clinical chemistry that a revolution in analytical methodology has been underway. About 30 years ago, a technician in a then modern clinical laboratory could handle a work load of 30 to 50 specimen analyses per day. In the modern automated laboratories of today, analyses may be conducted at a rate of about 2400 per hour. Clearly the business of clinical chemistry is a dynamic one and, as in all dynamic businesses, new technology is continually introduced which improves and sometimes supplants the old. Emerging now is such a new technology, one which makes use of dry reagent carriers to simplify analytical protocols. In general, these devices are totally self-contained analytical elements. Their use requires only that specimens be deposited on them and that analyte activity be read directly from a measurement of a change in their optical properties. In this paper, some of the principles, advantages, and limitations of the dry reagent analytical elements are described.

### Approaches to dry chemistry

Although several different groups are active in the development of this technology, there appear at present to be just two fundamental approaches. Each, apparently, is based on adaptation of the developing organisation's own technical specialty. Thus, one approach makes use of multilayered films similar in configuration to photographic film, while the second approach is an adaptation of the now well known dip stick technology for urinalysis; the analytical reagents are impregnated into paper fibres. In both systems, measurements are made of light reflected from the carriers following their exposure to specimen, and workers using both approaches make reference to measurement of reflectance. Perhaps because of the common usage of that term, the two different approaches appear to be regarded as technically identical. In fact, the term "reflectance" has a somewhat different meaning to each of the schools working on these systems, and the physical foundations of measurement of each are distinctly different. These differences lead to different analytical constraints as well as to different advantages.

In order to discuss the two systems, it is important to establish some definitions. First, there are two different kinds of reflection that one can describe; these are illustrated in Figure 1. Specular reflection, that illustrated in Figure 1(a), is mirror reflection. The angle of incidence equals the angle of reflectance. If the reflecting material is transparent, then some of the incident light will also pass into it and be refracted, that is, bent as it passes through the interface; and the bend angle will be related to the ratio of the indicies of refraction of the two media separated by the interface.

In Figure 1(b) a diffuse reflector is shown, the physical analogy of which might be a matte white wall lighted by the sun. The characteristic feature of a diffuse reflector is that light is reflected from its surface in a hemispherical distribution which is independent of the angle of incidence. Multilayer film technology makes use of a combination of both diffuse and specular reflection while impregnated fibre technology depends only on diffuse reflectance.

*Paper presented at the Analytica 80 symposium on Dry Reagent Chemistry in Munich, 29 April 1980.*

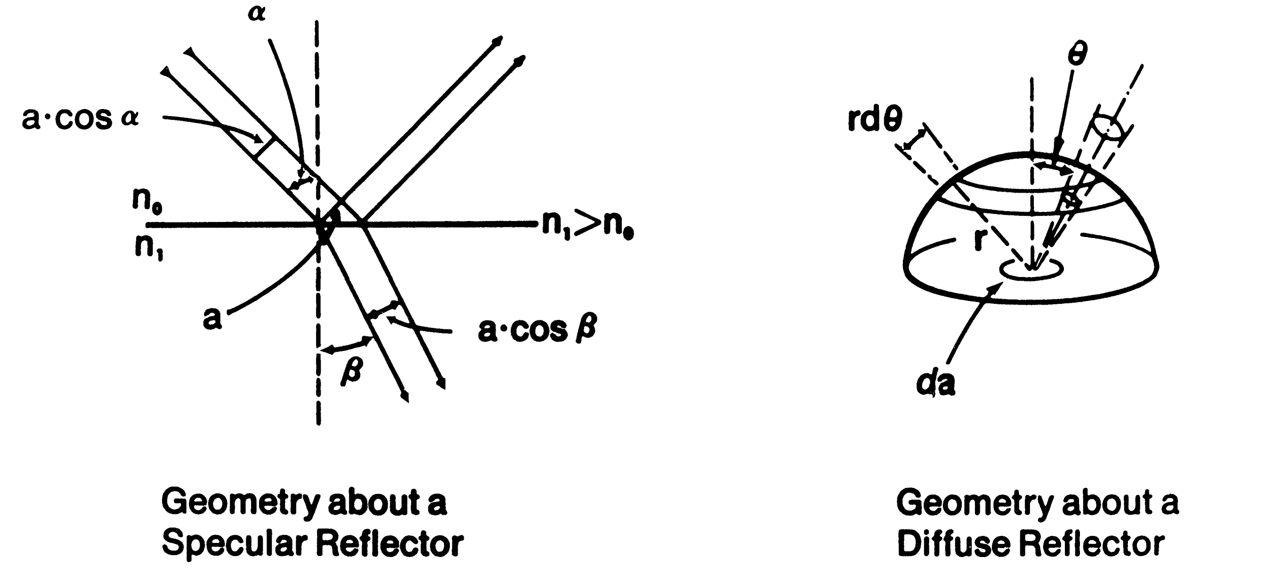

*Figure 1. Schematics of specular and diffuse reflectors*

Reflectance from a diffuse reflector is usually given by the ratio of the signal coming from the reflecting surface to the incident signal causing the reflection, i.e.,

$$\% R = \frac{I_{reflected}}{I_{incident}} \times 100 \qquad \text{..........} (1)$$

For multilayer reflection, the term reflectance density is used. It is a term taken from photographic science and is defined in terms of the inverse log of reflectance, i.e.,

$$D_R = \log \frac{I_{incident}}{I_{reflected}} \qquad \text{..........} (2)$$

### Theory of application

Of the several analytical approaches that are available for quantitative analysis of simple diffuse reflectance, probably the best known is the phenomenological theory of Kubelka and Munk derived in the early 30's [1]. The basis of that theory is illustrated in Figure 2. The drawing is a simple model for a reflecting medium with a backing material of reflectance $R_g$. As light passes downward through the medium, some of it is absorbed and some of it is scattered. Light that isn't absorbed ultimately reaches the backing layer where it is reflected back again. As it travels back up, more of it is absorbed and more is scattered. Light scattered from the downward directed beam adds to the intensity of the light directed upward and vice-versa, according to the model.

Equations 3 and 4 describe the changes in the intensity of the upward and downward directed light.

$$dI_+ = -(S + K)I_+dx + SI_-dx \qquad \text{..........} (3)$$

$$dI_- = -(S + K)I_-dx + SI_+dx \qquad \text{..........} (4)$$

where:

S = Scattering coefficient

K = $\epsilon$C = Absorption coefficient

$\epsilon$ = Extinction coefficient

C = Concentration

The first term on the right hand side of Equation 3 expresses the loss of light in the downward direction due to scattering and absorption. The second term represents the light gained in the downward direction due to the scattered light from that moving in the upward direction. Equation 4 is similar and simply describes, in the same way, the upward moving light. The constants, K and S, are proportionality factors for absorption and scattering, respectively. Similar to conventional absorption spectroscopy, K can be equated to the product of an effective extinction coefficient and the concentration of absorbing species. The value of K, of course, is wavelength dependent. The scattering coefficient may also be wavelength dependent; however, practically speaking, once wetted, the impregnated fibres that have been experimented with exhibit a scattering coefficient that appears to be essentially constant. It may be interesting to note, incidentally, that for a scattering coefficient equal to zero, which would imply a transparent medium, the Kubelka-Munk equations reduce to the derivative form of the Beer-Lambert law.

The solution to the differential equations shown in Figure 2 is given in Equation 5.

$$R = \frac{(R_gR_\infty)R_\infty(R_g - \frac{1}{R_\infty}) R_\infty \exp [SX (\frac{1}{R_\infty} - R_\infty)]}{R_g - R_\infty - (R_g - \frac{1}{R_\infty}) \exp [SX (\frac{1}{R_\infty} - R_\infty)]} \qquad \text{...} (5)$$

As can be seen, diffuse reflectance is a complicated function of the background reflectance, $R_g$, the scattering coefficient, S, the thickness of the reflecting medium, X, and a term denoted $R_\infty$. It is through $R_\infty$ that one can relate reflectance to the value of K and, thus, concentration. $R_\infty$ is the reflectance of a medium of effectively infinite thickness. In the case of paper, this thickness is somewhat less than a millimetre. It can be shown that for the special case of infinite thickness, $R_\infty$ becomes a function of K and S only, as is shown in Equation 6.

$$\frac{(I-R_\infty)^2}{2R_\infty} = \frac{K}{S} = \frac{\epsilon C}{S} \qquad \text{..............} (6)$$

Equations 5 and 6 are the diffuse reflectance spectroscopy analogs to Beer's law and they are used in a similar fashion in analytical applications.

Figure 3 illustrates a schematic of a multilayer film, dry reagent test. The test is composed of a pigmented spreading layer at the top; this is also a light reflecting layer. Immediately beneath is a subbing layer, that is, a layer that helps adhesion between the spreading layer and the gel layer beneath. The gel layer contains the reactive components of the test system. The individual layers are cast on a transparent film base. The specimen is placed on the spreading layer and diffuses down to the reacting layer. Reflectance is measured from below; that is, light incident on the film

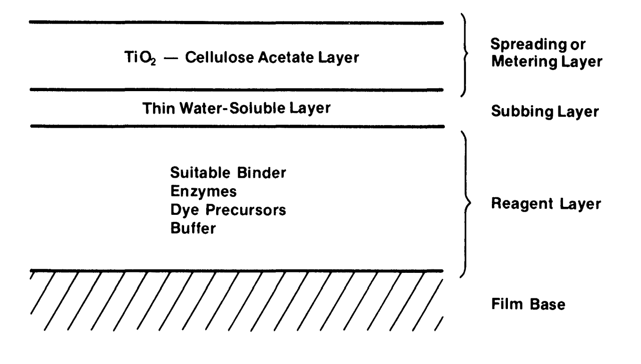

*Figure 3. Basic multilayer film configuration*

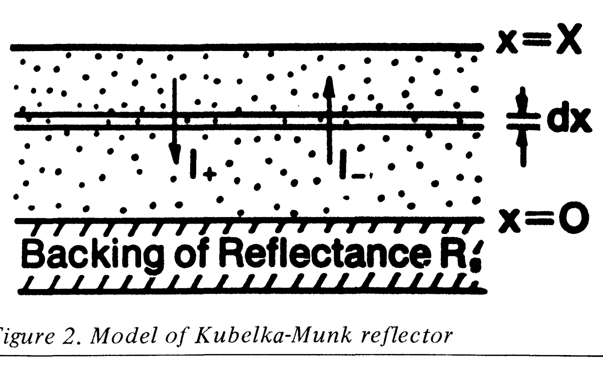

*Figure 2. Model of Kubelka-Munk reflector*

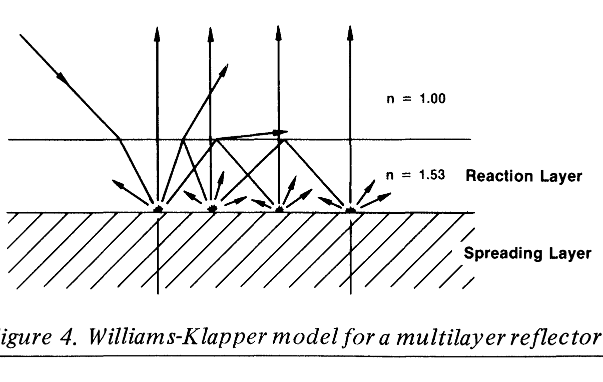

*Figure 4. Williams-Klapper model for a multilayer reflector*

base passes up through the reagent layer and is diffusely reflected from the spreading layer. Detection is through a light sensor oriented normal to the film base.

An optical analog of the multilayer film tests is illustrated in Figure 4. The shaded area in the figure corresponds to the spreading layer. A transparent area is shown with a refractive index of 1.53, that of gelatin. It corresponds to the reagent layer. The film base is not included to simplify the mathematical treatment. It is assumed that light passes from air through the reagent layer to the spreading layer. In the multilayer film test, light is incident at a 45 degree angle.

Light incident on the surface of the test slide will be specularly reflected, but some will pass into the reagent area. Because of the difference in index of refraction between air and the reagent area, there will be some refraction at the interface. The light will be bent to a shallower angle and pass through the reagent layer until it strikes the spreading layer. Some of the light will be absorbed in the reagent layer according to Beer's law, and some will be diffusely reflected by the spreading layer. Light reflected back through the reagent layer that is not directed normal to the interface will be re-reflected specularly by the interface and will again pass through the reagent layer. This process wil repeat in multiple reflections until the remaining light exists normal to the interface or is totally absorbed by its transit through the reagent layer.

The mathematical treatment of this kind of system is complicated. It involves consideration of diffuse reflectance, ordinary transmittance, and specular reflectance of hemispherically distributed incident light rays. However, Williams and Clapper [2] of the Eastman Kodak research laboratories were able to construct a mathematical model of the system, which is shown in Equation 7.

$$D_R = \log\frac{R_o}{R_{test}} = -\log\left\{0.193t^{2.13}\left[\frac{1}{2R_B} - \int^{\pi/2} t^{2\sec\theta} r_\theta \sin\theta\cos\theta\, d\theta\right]^{-1}\right\} \quad (7)$$

where :

$t$ = Film transmittance

$R_B$ = Spreading layer reflectance

$r_\theta$ = Interface reflectance

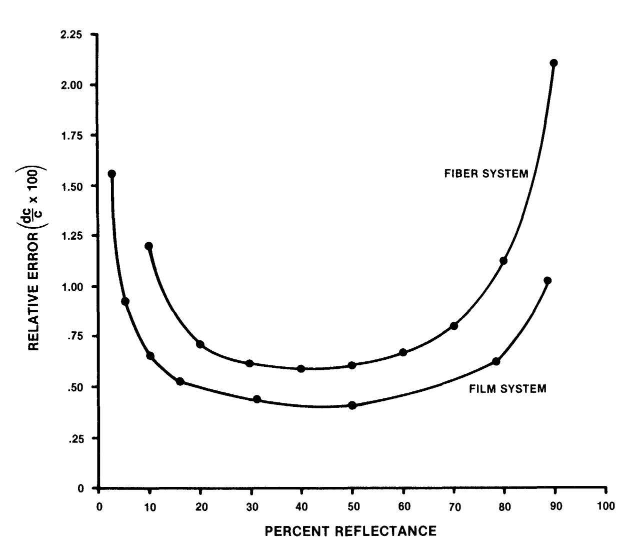

*Figure 5. Theoretical error resulting from 0.1% reading error*

As can be seen, reflection density, as defined earlier, is cast in terms of film transmittance, spreading layer reflectance, and the specular reflectance of the interface. This complicated integral function, like the Kubelka-Munk differential equations, lends itself to solution such that one can relate reflection density to concentration directly. That relationship is shown in Equation 8 and can be seen to include terms for an effective absorbance, $\beta$, analogous to the Kubelka-Munk K, and a blank reflection density, $D_b$.

$$C = \beta\left\{\left[-0.194 + 0.469D_R + \frac{0.422}{1 + 1.179e^{3.379D_R}}\right] - D_b\right\} \quad (8)$$

Equations 6 and 8 may differentiated with respect to reflectance and, with some manipulation, each can be cast into an expression for relative measurement error. Equations 9 and 10 result and may be used to compare the theoretical precision limits of the multilayer films with those of the impregnated fibres.

$$\frac{dC}{C} = \frac{(R_\infty + 1)}{(R_\infty - 1)R_\infty} \cdot dR_\infty \quad \ldots\ldots\ldots\ (9)$$

$$\frac{dC}{C} = \frac{\left\{0.469 - \dfrac{1.681e^{3.379D_R}}{\left[1 + 1.179e^{3.370D_R}\right]^2}\right\}}{\left\{-0.194 + 0.469D_R + \dfrac{0.422}{\left[1 + 1.179e^{3.379D_R}\right]}\right\}} \cdot \frac{dR}{2.3R} \quad \ldots(10)$$

Those limits are illustrated in Figure 5. The curves shown are based on assumption of reflectance colorimeter readability of about 0.1%, a value found to be practically achievable.

As can be seen, over the entire range of reflectance, multilayer films exhibit somewhat better theoretical

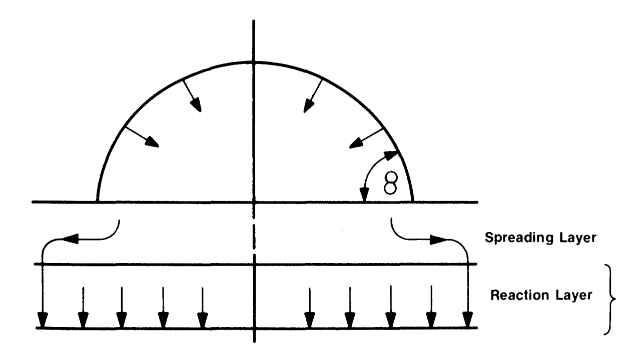

*Figure 6. Schematic of the spreading process – multilayer film test*

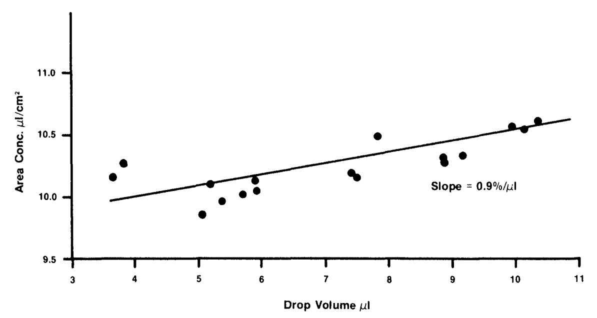

*Figure 7. Area concentration vs fluid volume – multilayer film test*

precision than impregnated fibres. It should be emphasised that neither system is bad; both are capable of yielding precision values of less than 1%, which is certainly clinically acceptable. The film system, however, has better precision over a somewhat broader range of reflectance. What this implies is that the potential measurement resolution of the film system may be somewhat better than that of the impregnated fibres.

A disadvantage of the film system that becomes evident in analysis of its theoretical model, however, is that its dose response character is distinctly non-linear. It requires a three point calibration [3]. In the case of diffuse reflectance, and application of Kubelka-Munk theory, linear response regions can be developed, and a two point calibration is all that is required.

That these two systems are distinctly different is quite evident from consideration of the two physical models. Their differences, however, extend beyond different system configurations and error limits. The configurational differences give rise to different constraints in the way they are used, the chemistry that may be adaptable to them, and even to some of their behavioural characteristics.

### Practical differences between two systems

Referring to the basic multilayer film configuration (Figure 3), it is noted that the specimen is placed on top of the spreading layer. In the system currently available, a specimen volume is used which is not sufficiently large to saturate the entire test area. Placement of the specimen droplet on the spreading layer initiates a process which is illustrated in Figure 6 [4]. The droplet first rapidly wets the spreading layer in a lateral direction and then diffuses down into the reaction layer. Larger droplets simply spread over larger areas and react with wider portions of the reaction layer. The colour is generated over a larger area of the test slide, but since only a small central area of the slide is observed, the measured response becomes nearly independent of volume. The extent to which volume independence is exhibited is illustrated in Figure 7 [4] in which a term called area concentration versus drop volume is plotted. Area-concentration may be equated to a test response per unit test area. There is a very low slope, that is to say, the area concentration of the specimen is almost independent of the drop volume.

In contrast to this rather attractive characteristic of the multilayer film test systems, impregnated fibre responses depend upon specimen volume. The reason for this is the fact that impregnated fibre tests must be saturated with specimen. Thus, end point tests, in particular, yield responses proportional to added volume; this is illustrated in Figure 8, which shows the apparent uric acid level of a uric acid strip test as a function of volume added. Based on these data, it can be seen that an error of about one third of a milligram per decilitre per microlitre could be expected. It should be emphasised, however, that with modern pipet construction, deposition of a 30 μl specimen, which is typical for an impregnated fibre test, should result in errors of less than 1 μl. Thus, pipetting errors, in general, will be lower than the expected overall error of the entire test system.

Volume dependence appears in the impregnated fibre systems in rate tests as well as end point tests, but to a lesser degree; this is illustrated in Figure 9 which shows apparent glucose value as a function of pipetted volume. Again, it can be seen that the errors are quite small, about 1.5 mg%/μl, and lower than the generally expected precision of the glucose test itself.

Countering the advantage the multilayer film format has with respect to specimen volume independence is its potential sensitivity to specimen viscosity or surface tension. Only for specimens of equal viscosity and similar surface tension will area concentration be independent of specimen volume. For specimens with large differences in viscosity lateral spreading characteristics in the spreading layer will not be the same. The consequences of viscosity are illustrated in Figure 10 [5] which shows the influence of increasing protein concentration on the apparent glucose value obtained from a multilayer film test. The increasing protein concentration, which also increases viscosity, results in differences in apparent glucose level of as much as 25%. Thus the range of protein level is broader than one would expect in a clinical situation. The data are shown for illustrative purposes and to point out that normalising to constant spot size, which effectively removes viscosity dependence, eliminates a great part of the effect; this is seen in the upper curve.

Because the impregnated fibre tests are saturated with specimen, their responses are essentially independent of specimen viscosity; this is illustrated in Figure 11, which shows apparent glucose value as a function of specimen protein concentration. As can be seen, the effect of viscosity is negligibly small compared to that observed with the multilayer film system. In Figure 12, similar data are shown for an impregnated fibre BUN assay. Again, viscosity is seen to have little effect on the apparent BUN value.

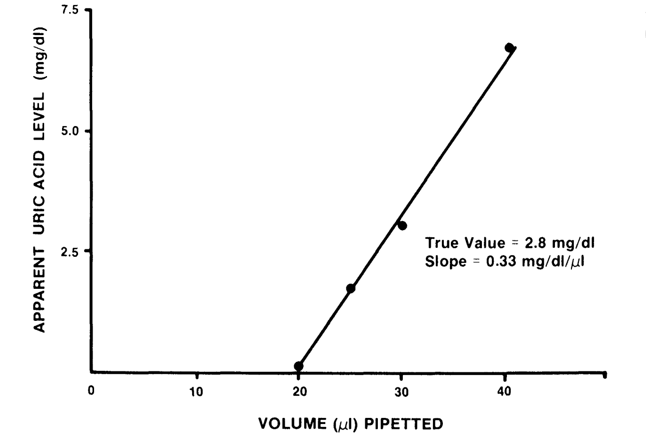

*Figure 8. Effect of variation in sample volume on an impregnated fibre end point test. All data are relative to calibration volume of 30 μl*

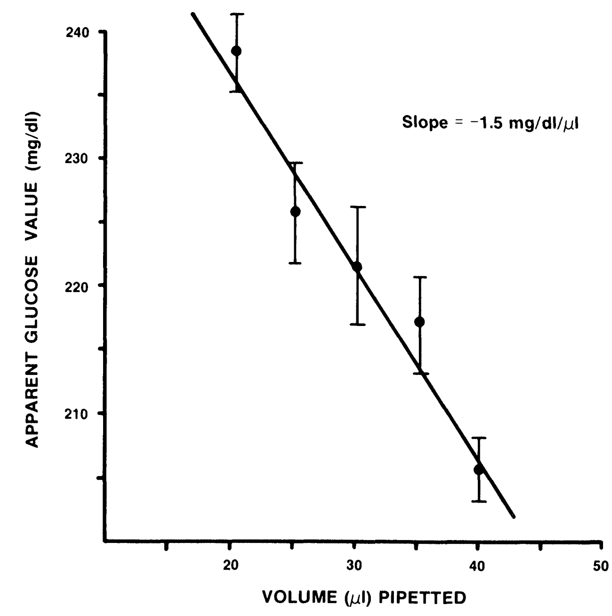

*Figure 9. Effect of variation in sample volume on an impregnated fibre rate test. Data are relative to a calibration volume of 30μl.*

The physical configurations of the two approaches lead to some other differences in physical characteristics, implied by reference to Figure 3. It is to be noted that the spreading layer is pigmented with titanium dioxide, an efficient light absorber at 340 nm. Thus, it is doubtful that this multilayer film configuration will ever be utilised for ultraviolet analysis, as for example, for tests like LDH or AST. Assays for these enzymes will be required that will allow monitoring their activity in the visible region of the spectrum. It may be possible to substitute an ultraviolet reflecting pigment for the titanium dioxide. However, the use of $TiO_2$, which is an extraordinarily efficient light scattering material, suggests that its substitution could result in significant light transmission losses through the 100 micron thick spreading layer, reducing the overall measurement sensitivity of the system.

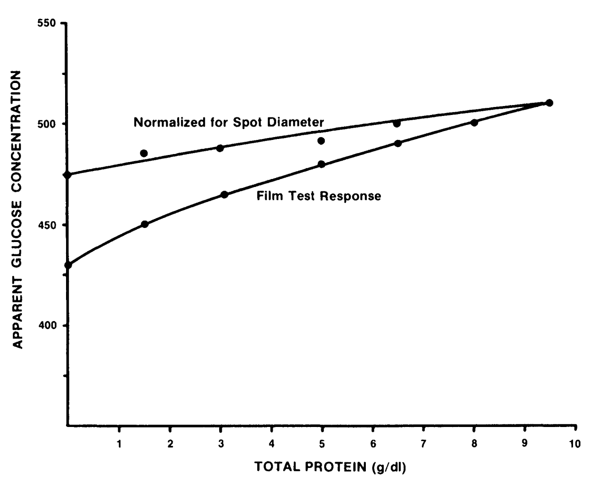

*Figure 10. Effect of protein on apparent glucose response of a multilayer film test*

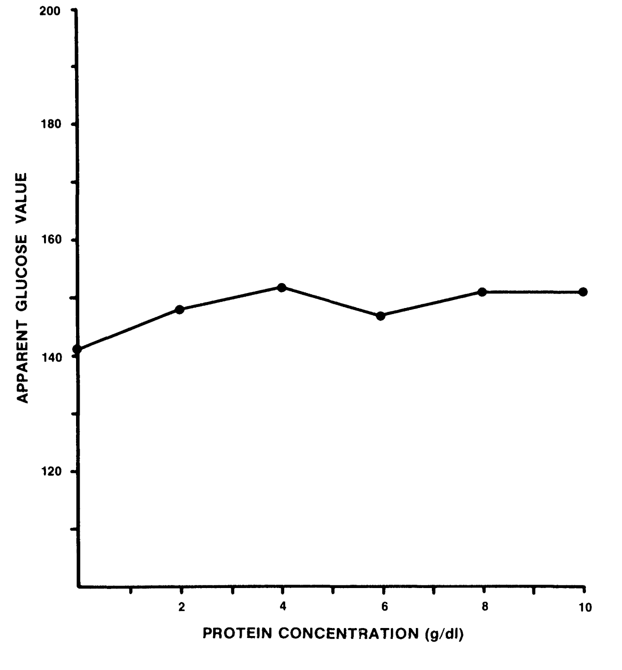

*Figure 11. Effect of protein on the glucose impregnated fibre assay*

Although the possibly narrow spectral breadth of the multilayer film tests is more a development than a user problem, it is still worthwhile emphasising that impregnated fibre tests may be used for conducting conventional ultraviolet enzyme assays. This is advantageous in two respects. First, dry reagent tests for ultraviolet assays may be able to be produced in a more timely fashion with impregnated fibre systems than with multilayer film systems. Second, conventional enzyme chemistry, which can be incorporated in a relatively straightforward way into impregnated systems, may be more acceptable to clinical chemists than any radically new chemistry which the film systems may require.

Another point worth mentioning relates to the multilayer film reaction layers that have been reported to date; these are, in general, made of gelatin. The use of gelatin precludes extremes of pH. Thus, for the multilayer film bilirubin assay, as reported in the patent literature [6], a wholly new approach was developed, which allowed the analysis to be carried out at neutral pH levels. It has been possible, on the other hand, to incorporate all the elements of a standard azo dye coupled bilirubin assay into an impregnated fibre system, including buffering to a pH level somewhat lower than 1, and simultaneously retain long term room temperature storageability for the test.

Test storageability is, in general, a significiant advantage of impregnated fibre systems in contrast to multilayer films. The gelatin matrix of the multilayer films gives rise to a requirement for refrigerated storage in well sealed packages. Unless refrigerated, the tests are said to have limited lifetime, and unsealed packages have been reported to degrade rapidly, refrigerated or not. The limited life of the films probably derives from residual water in the gelatin matrix, which allows for liability of the incorporated bioreagents. Aqueous gels containing proteins are no more stable than aqueous solutions of proteins, as all who have done immunodiffusion analysis are well aware. Impregnated fibre systems contain little residual water and exhibit extraordinary stability without need for refrigeration or sophisticated packaging methods. The paper strip tests are packaged in ordinary glass or plastic jars and, in a conventional laboratory environment, can be opened and closed numerous times without significant degradation of the contents.

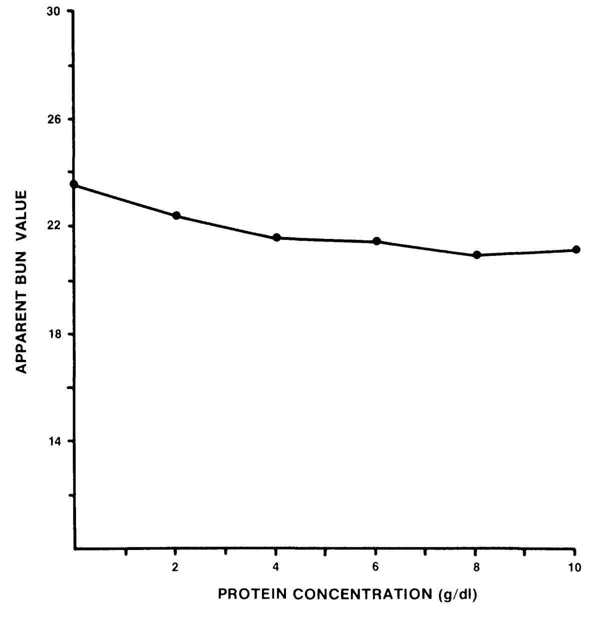

*Figure 12. Effect of protein on the BUN impregnation fibre assay*

### Conclusions

It appears that on balance, these two systems are similar in terms of advantages and disadvantages. Each seems to have emphasised the more important characteristic for its potential application. Thus, the multilayer film tests seem to be being directed to large laboratories where high precision is important and storageability less so. The impregnated fibre systems, on the other hand, seem to be being directed toward the small laboratory, the physician's office, or the emergency room, where convenience, low space requirements, and simplicity are important. Unquestionably, as dry reagent technology matures, each will borrow from the other to achieve the best features of both. One should bear in mind that the technologies, as they are currently configured, are only the beginning. They may, however, signal the future of clinical chemistry. If successful, they certainly will bring a new and desirable level of analytical standardisation to the industry and provide the medical community with a more universal basis for communication. Large scale manufacturing with its associated quality control methods should provide a new level of analytical consistency. Laboratory regulation should become simplified. Finally, the new technology should enhance cost effectiveness in health care because it provides capability for test selectivity, because of its ease of use, and because individual self-contained tests allow for little material waste.

REFERENCES

[1] Kubelka P. and Munk F., Z. (1931) *Tech. Physik.*, **12**, 593
[2] Williams F. C. and Clapper F. R., (1953) *J. Opt. Soc. Amer.*, **43**, 595
[3] Curme, H.G., et al, (1978) *Clin. Chem.*, **24**, 1335.
[4] Eder T. W., Abst., X Intl. Cong. Clin. Chem., Mexico City, Feb., 1978
[5] McGlothlin, C.D., Abst. No. 375, 30th Natl. Mtg., A.A.C.C., San Francisco, July, 1978
[6] Wu,T. and Dappen, M., U.S. Patent 4,069,017

### Development of dry reagent chemistry for the clinical laboratory*

Adam Zipp
*Blood Chemistry Laboratory, Ames Divison, Miles Laboratories, Eikhart, Indiana, USA.*

### Introduction

The Ames Division of Miles Laboratories has, for the past several years, been actively involved in the development of a quantitative serum chemistry system. Initially, the system will consist of the instrument, a reflectance photometer called the Seralyzer, along with five solid phase reagents: glucose, BUN, uric acid, bilirubin, and LDH. Reagents to analyse for cholesterol and triglycerides are also under development. The technology necessary for the development of this system stems from the development by Ames of urine strip products. The knowledge obtained from the development of the Dextrostix/Eyetone system for the quantitative analysis of whole blood glucose also contributes. The company has however, extended this technology to include a host of serum metabolites as well as enzymes. A schematic of the solid phase reagent strip is shown in Figure 1. This strip contains a cellulose matrix into which is impregnated those reagents necessary for a given clinical chemical determination. After the impregnation process, the matrix is dried and bonded with a special adhesive layer onto a plastic support which allows for ease of insertion onto and removal from the instrument. The philosophy adopted during the development of this system is to employ well-known methodologies wherever possible. The use of cellulose provides a good deal of flexibility in this regard so that it is possible to impregnate a host of different materials into the matrix, even under extreme conditions. For example, serum bilirubin is quantitated with a diazonium coupling reaction which is carried out at pH near 1. Similarly, the solid phase BUN test is carried out in a cellulose matrix which is a 50% cation exchange resin so that very low pH conditions are also achieved here.

*Paper presented at the Analytica 80 Symposium on Dry Reagent Chemistry in Munich, April, 1980.*

*Figure 1.*

